# Language Evolution

**DOI:** 10.1371/journal.pbio.0020346

**Published:** 2004-10-12

**Authors:** Szabolcs Számadó, Eörs Szathmáry

## Abstract

How did language develop and evolve? Here, linguists, cognitive scientists, behavioural ecologists, and theoretical biologists all offer their disparate views on this emerging field

A ban in the 1866s by the French Academy of Sciences on publications about the origin of human language must have been one of the strangest bans in the history of sciences. Yet it was highly effective. After the ban, scientists and interested laymen had to wait for more than a century to hold a textbook on language evolution in their hands. *Language Evolution,* a compilation of essays by a diverse group of respected researchers, is amongst the first books that try to tackle what is arguably one of the hardest scientific problems. The editors set themselves the ambitious target of creating an up-to-date book about this emerging field, and they have to be congratulated for their efforts. Linguists, cognitive scientists, behavioural ecologists, and theoretical biologists all offer their view on the origin of human language and, refreshingly, do not shy from pointing out the real or assumed weaknesses of the other approaches.[Fig pbio-0020346-g001]

**Figure pbio-0020346-g001:**
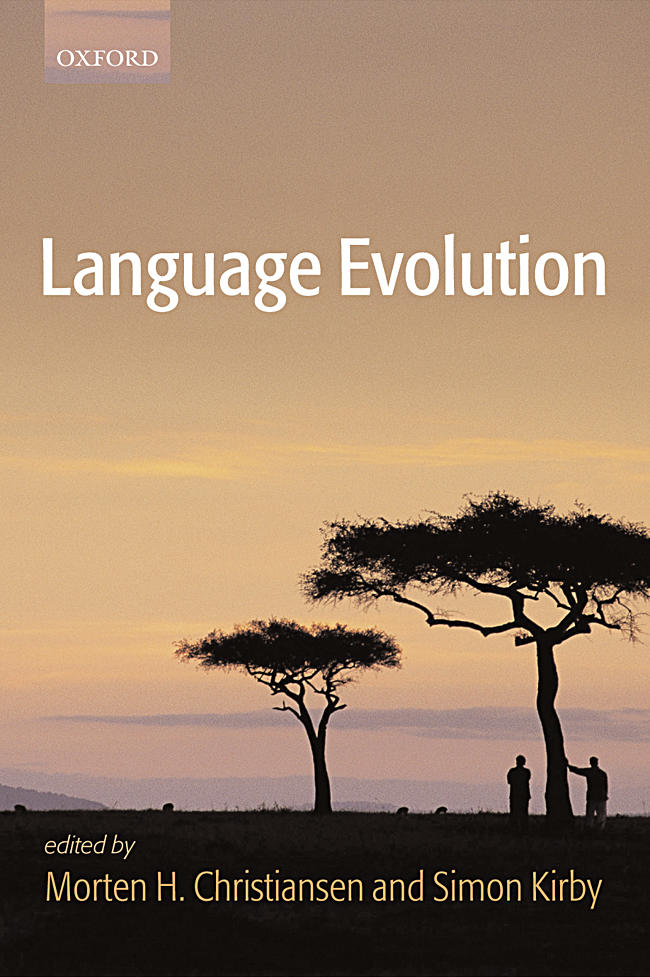


One of the main themes of the book is the evolutionary approach and the importance of biological structures and properties that were co-opted in the development of language (pre-adaptations). In one essay, Michael Studdert-Kenedy and Louis Goldstein propose that speech, as a motor function, draws on phylogenetically ancient mammalian oral capacities for sucking, licking, swallowing, and chewing. Thus, our hominid ancestors adopted an apparatus already divided neuroanatomically into discrete components. Complementing this evidence, Marc Hauser and Tecumseh Fitch compare human speech production and perception with that of nonhuman species. They conclude that many traits that were formerly thought to have evolved specifically for speech (such as having a descended larynx or categorical perception) are also present in other species.

But perhaps the most interesting idea about pre-adaptation comes from the work of neuroscientist Michael Arbib on ‘mirror’ neurons in monkeys. These neurons are a subset of the grasp-related premotor neurons that discharge not only, as other premotor neurons do, when the monkey executes a certain class of actions, but also when the monkey observes more or less similarly meaningful hand movements made by the experimenter (or by another monkey). The area in which these grasp-related neurons are found is analogous with the Broca's area in human brains, which is involved in assessing the syntax of words. This observation serves as the basis for the mirror-system hypothesis, which postulates that Broca's area in humans evolved from a basic mechanism not originally related to communication but rather from the mirror system for grasping in the common ancestor of monkey and human. As a result, the mirror system provides a possible ‘neural link’ in the evolution of human language.

There is still much debate about the selection pressures that led to the evolution of language. Observing the overabundance of potential selective scenarios for why language evolved, the linguist Derek Bickerton voices his scepticism: ‘The fact that these and similar explanations flourish side by side tells one immediately not enough constraints are being used to limit possible explanations.’ One frequent source of confusion, he notes, is equating language with speech by not distinguishing between modality, lexicon, and structure. Hauser and Fitch share Bickerton's scepticism and urge scientists to rely more on the traditional comparative approach, which was always the strength of Darwinian evolutionary theory.

Primatologist Robin Dunbar, who originally proposed that grooming (group bonding) could have provided the stimulus for language, dismisses two other possible scenarios—hunting and tool-making—as potential ecological contexts for the evolution of human language. Gestural origins are also dismissed in his theory, because gestural languages do not seem to develop spontaneously and also require a line-of-sight contact making them useless at night.

Interestingly, Steven Pinker rules out both Dunbar's theory of grooming and Geoffrey Miller's theory of sexual selection, whereas Bickerton rules out grooming, gossip, mating contract, and Machiavellian intelligence as likely contexts for the origin of human language.

Also under fire in the book is the idea that the human brain is somehow equipped at birth with a ‘universal grammar’ out of which all human languages later develop. Several authors try to provide alternatives to innate predispositions, such as the importance of function to categorization (Michael Tomasello) and the importance of cultural transmission to the structure of language (Simon Kirby and Morton Christiansen). Arbib explicitly questions the traditional Chomskyan theory of innate linguistic predispositions and argues that what humans have and had in the past is ‘language readiness’ rather than a fixed universal grammar.

Neuroscientist Terrence Deacon also puts an alternative theory forward. According to Deacon, many of the language universals reflect semiotic constraints inherent in the requirements for producing symbolic reference rather than innate predispositions. Thus, neither evolved innate predispositions nor culturally evolved and transmitted regularities can be considered as the ultimate source of language universals. He draws a parallel with mathematical operations (addition, subtraction, etc.) and with prime numbers. Symbolic reference, he argues, is constrained by the structure it refers to.

The editors claim, in the light of this diversity, that ‘this book is intended to bring together, for the first time, all the major perspectives on language evolution’. We have two concerns with this aim. First, two books of the same organization and scope have been published in the past six years based on the material from language evolution conferences (Hurford et al. 1998; Knight et al. 2000). Although this first concern might be just splitting hairs, the second is more substantial: several crucial aspects of language evolution are not represented at all or are just touched superficially.

One of these missing themes is the selective advantage of early language. As discussed, many of the contributors express their scepticism towards the selective scenarios found in the literature—and indeed towards such constructions in general—but there is no review and no balanced evaluation of these selective scenarios. Since one of the key questions of language evolution is the selective advantage of early language, the lack of such a review is a major weakness. A balanced account could have been presented even if the editors and most of the contributors are frustrated by the plethora of selective scenarios.

Related to the possible selective advantage of language is the issue of genetic background. Although there is mention of the so-called FOX genes—some mutations of which are associated with language disorders—there is no detailed discussion of our current knowledge of genetics related to language.

Another lightly treated theme is the neural basis of language and language evolution. Understandably it is one of the most difficult issues concerning human language, and no one expects the editors or any of the contributors to come up with an answer to all the questions. What is missing again is a good survey outlining the problems and the current findings of the field.

The weaknesses of the book come from its structure and organization. The editors, instead of outlining a structure and asking specialists to contribute to that structure, appear to have let every contributor write freely about their current ideas and current research without regard to the bigger picture. This definitely shows the interests of the contributors and outlines the current state of the art; it leaves gaps, however, in the coverage of crucial topics related to the evolution of human language.
